# Rational Design of Layered SnS_2_ on Ultralight Graphene Fiber Fabrics as Binder-Free Anodes for Enhanced Practical Capacity of Sodium-Ion Batteries

**DOI:** 10.1007/s40820-019-0297-6

**Published:** 2019-08-03

**Authors:** Zongling Ren, Jie Wen, Wei Liu, Xiaoping Jiang, Yanheng Dong, Xiaolong Guo, Qiannan Zhao, Guipeng Ji, Ronghua Wang, Ning Hu, Baihua Qu, Chaohe Xu

**Affiliations:** 1 College of Aerospace Engineering, The State Key Laboratory of Mechanical Transmissions, Chongqing University, Chongqing, 400044 People’s Republic of China; 2 Pen-Tung Sah Institute of Micro-Nano Science and Technology, Xiamen University, Xiamen, 361005 People’s Republic of China; 3 College of Materials Science and Engineering, Chongqing University, Chongqing, 400044 People’s Republic of China; 4 MOE Key Laboratory of Low-grade Energy Utilization Technologies and Systems, CQU-NUS Renewable Energy Materials and Devices Joint Laboratory, Chongqing University, Chongqing, 400044 People’s Republic of China

**Keywords:** SnS_2_, Graphene fiber fabric, Binder-free electrode, Practical capacity, Sodium-ion batteries

## Abstract

Generally, the practical capacity of an electrode should include the weight of non-active components such as current collector, polymer binder, and conductive additives, which were as high as 70 wt% in current reported works, seriously limiting the practical capacity. This work pioneered the usage of ultralight reduced graphene fiber (rGF) fabrics as conductive scaffolds, aiming to reduce the weight of non-active components and enhance the practical capacity. Ultrathin SnS_2_ nanosheets/rGF hybrids were prepared and used as binder-free electrodes of sodium-ion batteries (SIBs). The interfused graphene fibers endow the electrode a porous, continuous, and conductive network. The in situ phase transformation from SnO_2_ to SnS_2_ could preserve the strong interfacial interactions between SnS_2_ and graphene. Benefitting from these, the designed binder-free electrode delivers a high specific capacity of 500 mAh g^−1^ after 500 cycles at a current rate of 0.5 A g^−1^ with almost 100% Coulombic efficiency. Furthermore, the weight percentage of SnS_2_ in the whole electrode could reach up to 67.2 wt%, much higher than that of common electrode configurations using Cu foil, Al foil, or carbon cloth, significantly highlighting the ultralight characters and advantages of the rGF fabrics for using as binder-free electrodes of SIBs.

**Electronic supplementary material:**

The online version of this article (10.1007/s40820-019-0297-6) contains supplementary material, which is available to authorized users.

## Highlights

- Layered SnS_2_ nanosheets/reduced graphene fiber (SnS_2_@rGF) hybrid fabrics were fabricated as binder-free electrodes.
- Ultralight rGF fabrics could increase the active materials loading to a high level of 67.2 wt% in the whole electrode, which is much higher than that of traditional slurry coating electrodes by using Cu or Al foil as current collectors.
- The practical capacity based on the weight of electrode could increase to as high as ~ 538 mAh g^−1^ exceeding to the slurry coating electrodes.

**Electronic supplementary material:**

The online version of this article (10.1007/s40820-019-0297-6) contains supplementary material, which is available to authorized users.

## Graphical abstract

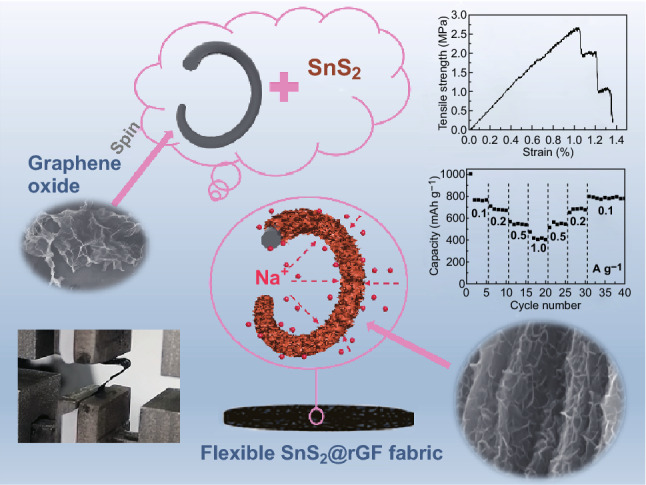

## Introduction

The development of clean energy is one of the center topics in sustainable society, because of the shortage of non-renewable energy resources as well as environmental pollution. However, the timeliness of current clean energy such as solar energy, wind energy, tidal energy calls for searching an efficient and inexpensive large-scale energy storage system with high energy density [1–3]. In recent years, room temperature sodium-ion batteries (SIBs) have been reported as a promising large-scale renewable energy storage system [4], on account of low cost and element abundance (~ 2.75% of sodium vs. ~ 0.065% of lithium) of the raw materials [5, 6]. However, the larger ionic radius and heavier atomic weight of Na result in sluggish kinetics and additional migration barrier energy of the electrode materials, which causes poor electrochemical reversibility and rate capability [7]. Thus, the searching of promising active materials for SIBs is greatly demanded for their future development and commercialization [8, 9].

Metal sulfides such as FeS [10], MoS_2_ [11–14], Sb_2_S_3_ [15], NiS_2_ [16], SnS_2_ [17, 18], and CoS_2_ [19] have been under intensive investigations in recent years because of their high theoretical capacity via conversion and/or alloying reactions with Na^+^ [17, 18, 20–22]. Typically, the SnS and SnS_2_ with large interlayer spacing and high theoretical capacity (about 700–1000 mAh g^−1^) and low operation potential are some of the most promising anode electrodes for SIBs [23–27]. However, the poor electrical conductivity and the huge volume expansion during Na^+^ insertion/extraction result in the pulverization of active materials and serious capacity fading, which extremely hinder the practical applications in SIBs [28]. In order to take the advantages, nanocarbons are employed to hybrid with SnS_2_ nanocrystals improving the conductivity and structural stability. Generally, reduced graphene oxide (rGO) is recognized as an ideal scaffold to accommodate the material pulverization and aggregation owing to its good mechanical property [7], high electrical conductivity, desirable flexibility as well as favorable chemical affinity with SnS_2_ materials, which has already been verified by many previous works [29–31]. However, SnS_2_/rGO-based electrodes were commonly prepared by a traditional slurry coating approach onto current collectors using inactive polymer binders (Table S1). The insulating nature of polymer binders will decrease the conductivity of the electrodes and block the ions diffusion [32–34]. Moreover, the real energy–density of the battery was significantly limited when considering the heavy mass of current collector and polymer binders. Thus, it is extremely required to develop new electrode configurations, such as binder-free electrodes with lightweight current collectors [35–37].

In most cases, carbon cloth was employed as general scaffolds to develop the binder-free electrodes for energy storage devices such as batteries and supercapacitors [38–42]. However, the challenge is that carbon cloth did not participate in the charge storage reactions but occupy very high mass contents (> 70 wt%), because the active materials could only decorate onto the limited outside surface of carbon fibers [25, 26]. Thus, searching for an ultralight material platform for binder-free electrodes with good mechanical and electrical properties is urgently demanded [35]. Very recently, the emerging of non-woven reduced graphene fiber (rGF) fabrics which consist of interfused graphene fibers possesses the desirable structure characteristics—an ultralight, flexible, robust, and highly conductive continuous network, thus providing a promising opportunity for the development of high performance binder-free electrodes for SIBs. Additionally, the intrinsic nature of GO offers the possibility for easy control of guest materials on rGFs. Moreover, the ultralight nature of rGFs is highly favorable for increasing the mass percentage of active materials to a high level. These performances make rGFs to be a potential scaffold to develop binder-free electrodes with high practical capacity [43]; however, the related study is quite limited.

Herein, through hydrothermal and sulfurization reactions, we firstly fabricated a SnS_2_@rGF hybrid textile as binder-free electrodes for SIBs by pioneering the usage of rGF as conductive scaffolds. Such electrodes possess several merits for using in SIBs: (i) The ultralight nature of rGF fabrics could significantly increase the effective mass loading of the active materials within the electrode (up to 67.2 wt%), thereby greatly enhancing the practical capacity compared to other electrode configurations using heavy Cu foil, Al foil or carbon cloth as current collectors; (ii) the highly porous rGF could not only provide rich of space, voids and channels for electrolyte ions immersion and diffusion, but also can accommodate the volume expansion of SnS_2_; (iii) the interfused graphene fibers can form a continuous conductive network and therefore endow rGF a high conductivity; (iv) SnS_2_ nanosheets are in situ transformed from SnO_2_ nanocrystals and thus could preserve the strong interfacial contact with graphene; and (v) the hybridization between SnS_2_ and rGF could inhibit Na_*x*_Sn aggregation during cycling. According to these advantages, the designed SnS_2_@rGF electrode shows a high utilization of active materials. The half cell could deliver a specific capacity of 836 mAh g^−1^ at 0.1 A g^−1^. Moreover, it provides a high capacity of 500 mAh g^−1^ after 500 cycles with Coulombic efficiency of ~ 100% at a high current density of 0.5 A g^−1^ The rGF is a promising framework for using as binder-free and ultralight electrode of SIBs with high practical capacity.

## Experimental Section

### Preparation of the SnS_2_@rGF Fabrics

Graphene oxide (GO) dispersion employed in this work was prepared by a modified Hummers approach. Briefly, 1.5 g expanded graphite (0.99, Qing Dao Teng Sheng Da Su Ji Xie Co., Ltd, Qingdao, China) and 1.15 g NaNO_3_ (AR, Chongqing Yi Bo Chemical Agent Co., Ltd, Chongqing, China) were carefully introduced into a beaker with 50.7 mL concentrated H_2_SO_4_ (Chuan Dong Chemical Agent Co., Ltd, Chongqing, China) and stirred for more than 2 h. Then the 6 g KMnO_4_ (AR, Chengdu Chron Chemicals Co., Ltd, Chengdu, Sichuan, China) was added into the beaker and stirred for 2 h. After stored for 3 days at ambient environment, 150 mL H_2_SO_4_ aqueous solution (5%) was dropped into the beaker under ice water condition; then 4.5 mL H_2_O_2_ (30%) (Chuan Dong Chemical Agent Co., Ltd, Chongqing, China) was added to the solution to stop the oxidizing reaction. The bright yellow product was washed with HCl solution (10%) (Chuan Dong Chemical Agent Co., Ltd, Chongqing, China) and then rinsed with deionized (DI) water to neutral via dialysis. After being centrifuged at 3000 rpm for half an hour to remove the impurities, the obtained GO dispersion was exfoliated by sonication and diluted to 6 mg mL^−1^ for spinning. GO fibers (GOF) was synthesized by a wetting spinning technique. 2.5 mL GO dispersion was injected into a rotating mixed coagulation bath (ethyl acetate and ethyl alcohol, 1:1 by volume, AR, Chengdu Chron Chemicals Co., Ltd, Chengdu, Sichuan, China) by a syringe to form GOF (1.5 mL h^−1^). The obtained long fibers were cut into short fibers with average length of ~ 10 mm. After vacuum filtration, GOF fabrics were collected and further dried at 60 °C for 6 h in vacuum oven.

The binder-free SnS_2_@rGF fabric electrode was fabricated by a two-step approach. Firstly, 0.7 g SnCl_4_·5H_2_O (AR, Chengdu Chron Chemicals Co., Ltd, Chengdu, Sichuan, China) was dissolved into a mixed solvent of DI water and ethylene glycol (AR, Guangdong Guanghua Sci-Tech Co., Ltd, Guangzhou, Guangdong, China) (60 mL, 1:1 in volume) by continuous stirring. After transferred into a 100 mL Teflon-lined stainless steel autoclave, a piece of GOF fabric was immersed into the precursor solution and heated at 150 °C for 12 h. After cooling to room temperature, the collected fabric (SnO_2_@rGF) was washed with DI water and absolute alcohol and dried in a vacuum oven for 6 h. Secondly, 0.5 g thioacetamide (AR, Chengdu Chron Chemicals Co., Ltd, Chengdu, Sichuan, China) was put into a ceramic crucible and covered by a stainless mesh. The SnO_2_@rGF fabrics were stacked on the top-side of stainless mesh and further treated at 400 °C for 3 h in Ar atmosphere (AR, Chongqing Ruixin Gas Co., Ltd, Chongqing, China) to get the SnS_2_@rGF fabrics. Pure rGF fabrics was synthesized under the same conditions without the presence of SnCl_4_·5H_2_O and thioacetamide. The SnS_2_ nanocrystals were synthesized under the same conditions as SnS_2_@rGF fabric but without the presence of GOF. Carbon cloth was firstly immersed into concentrated HNO_3_ (68 wt%) and kept at 60 °C for 1 h. Then, the treated carbon cloth was rinsed with numerous purified water and ethanol and further dried in vacuum oven. The SnS_2_@carbon cloth was synthesized under the same conditions as SnS_2_@rGF fabric.

### Material Characterizations

The crystal structures of products were analyzed by X-ray diffraction (XRD, Rigaku D/max 2500). Scanning electron microscopy (SEM) was taken with Zeiss AURIGA FIB. Transmission electron microscopy (TEM) measurements were conducted on a Zeiss LIBRA 200 FEG TEM with the operation voltage of 200 kV. X-Ray photoelectron spectroscopy (XPS) was performed on a KRATOS AXIS DLD spectrometer. Raman spectrum was collected on HORIBA LabRAM HR Evolution. Weight percentage of SnS_2_ was analyzed with thermogravimetric analysis on NETZSCH STA449C.

Electrochemical tests were carried out via a CR2032 coin cells assembled in an Ar-filled glovebox with H_2_O and O_2_ < 0.5 ppm. The as-obtained binder-free SnS_2_@rGF fabrics were used as the working electrode directly, with the sodium foil (Na metal, CP, 98%, Aladdin, Shanghai, China) served as counter electrode and a Whatman Glass fiber D as separator. The mass loading of SnS_2_ is 1.0–1.5 mg cm^−2^. The electrolyte was 100 µL 1 M of NaClO_4_ in a mixture of ethylene carbonate and diethyl carbonate (1:1 v/v) along with 5 wt% of fluoroethylene carbonate (AR, Shenzhen Kejingstar technology Co., Ltd, Shenzhen, Guangdong, China) in each cell. The controlled traditional SnS_2_ anodes were prepared as follows: SnS_2_ powders, polyvinylidenefluoride (AR, Chengdu Chron Chemicals Co., Ltd, Chengdu, Sichuan, China), and acetylene black (AR, Shenzhen Kejingstar technology Co., Ltd, Shenzhen, Guangdong, China) were mixed with weight ratio of 8:1:1 at first, followed by adding *N*-methyl-2-pyrrolidone (AR, Chengdu Chron Chemicals Co., Ltd, Chengdu, Sichuan, China) to form a homogenous slurry. The slurry was coated onto the copper foil and dried in a vacuum oven at 120 °C overnight. The mass loading of pure SnS_2_ electrode is 1.5 mg cm^−2^. The areal density of rGF is about 0.8–1.2 mg cm^−2^.

Galvanostatic charge and discharge measurements were carried out at different current densities at the voltage range from 0.01 to 3.0 V on a Neware battery tester. Cyclic voltammetry (CV) was evaluated with CHI 760E electrochemical workstation at a scan rate of 0.25 mV s^−1^ between 0.01 and 3.0 V at room temperature. Electrochemical impedance potential was tested at a frequency between 0.01 Hz and 100 kHz. All the capacities were calculated based on the weight of SnS_2_.

The conductivity of the GOF, rGF, and SnS_2_@rGF was tested on a digital multi-meter (VICTOR 86E, KEYSIGHT, Palo Alto, USA). The tensile strength was tested on a universal testing machine (UTM, Shimadzu, EZ-LX). The size of samples is 20 × 5 mm^2^, and the thickness is 14 µm. The tensile velocity is 0.05 mm s^−1^.

## Results and Discussion

**Fig. 1:**
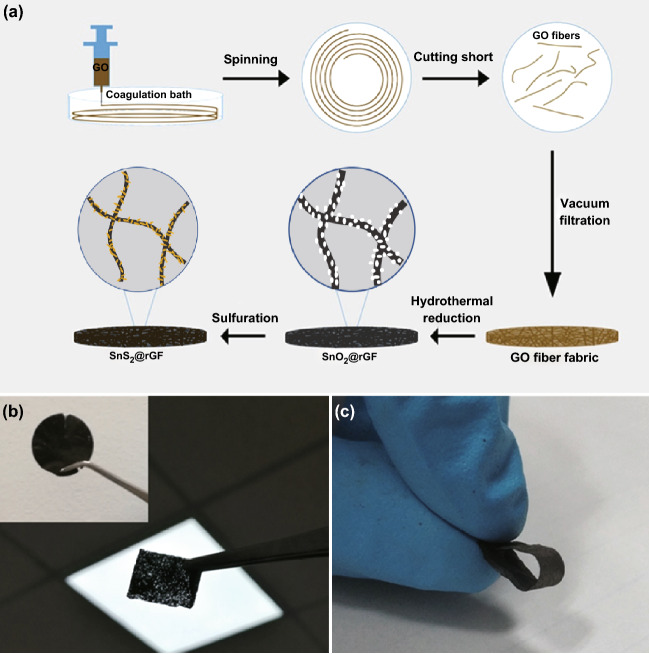
**a** Schematic illustration of the preparation process of the SnS_2_@rGF fabrics. **b, c** Photo images of the SnS_2_@rGF fabrics

Figure 1a shows a schematic illustration of the preparation process of the SnS_2_@rGF fabrics, including liquid crystal wet-spinning of GO fibers, self-assembling of rGF, and compositing of rGF with SnS_2_. Without the addition of any binders, GO dispersion with a concentration of 6 mg mL^−1^ and sheet sizes of > 50 µm (in Fig. S1a) could self-assemble into a continuous GO fiber [44]. After cutting into short fibers and vacuum filtration, highly porous and flexible GOF fabrics can be easily obtained (in Fig. S1b, c). Compared with other commonly used substrate, such as carbon cloth (Fig. S3), the present GOF fabric is not only ultralight but also rich in wrinkles and macropores (Fig. S1), which is highly beneficial to increase the mass loading of the active materials. Additionally, the intrinsic nature of GO-abundant of functional groups offers the possibility for easy control of nanocrystals on rGFs. The good performances suggest that as-prepared GOF fabrics could be a fascinating candidate of electrode. As a demonstration, ultrafine SnO_2_ nanocrystals were uniformly and in situ grew onto the rGF fabrics by a solvothermal reaction, without any particle aggregations (Fig. S4b, c). After subsequent sulfuration, the SnO_2_ nanocrystals were in situ transformed to SnS_2_ nanosheets and form a flexible SnS_2_@rGF fabric, as shown in Fig. 1b, c.

**Fig. 2:**
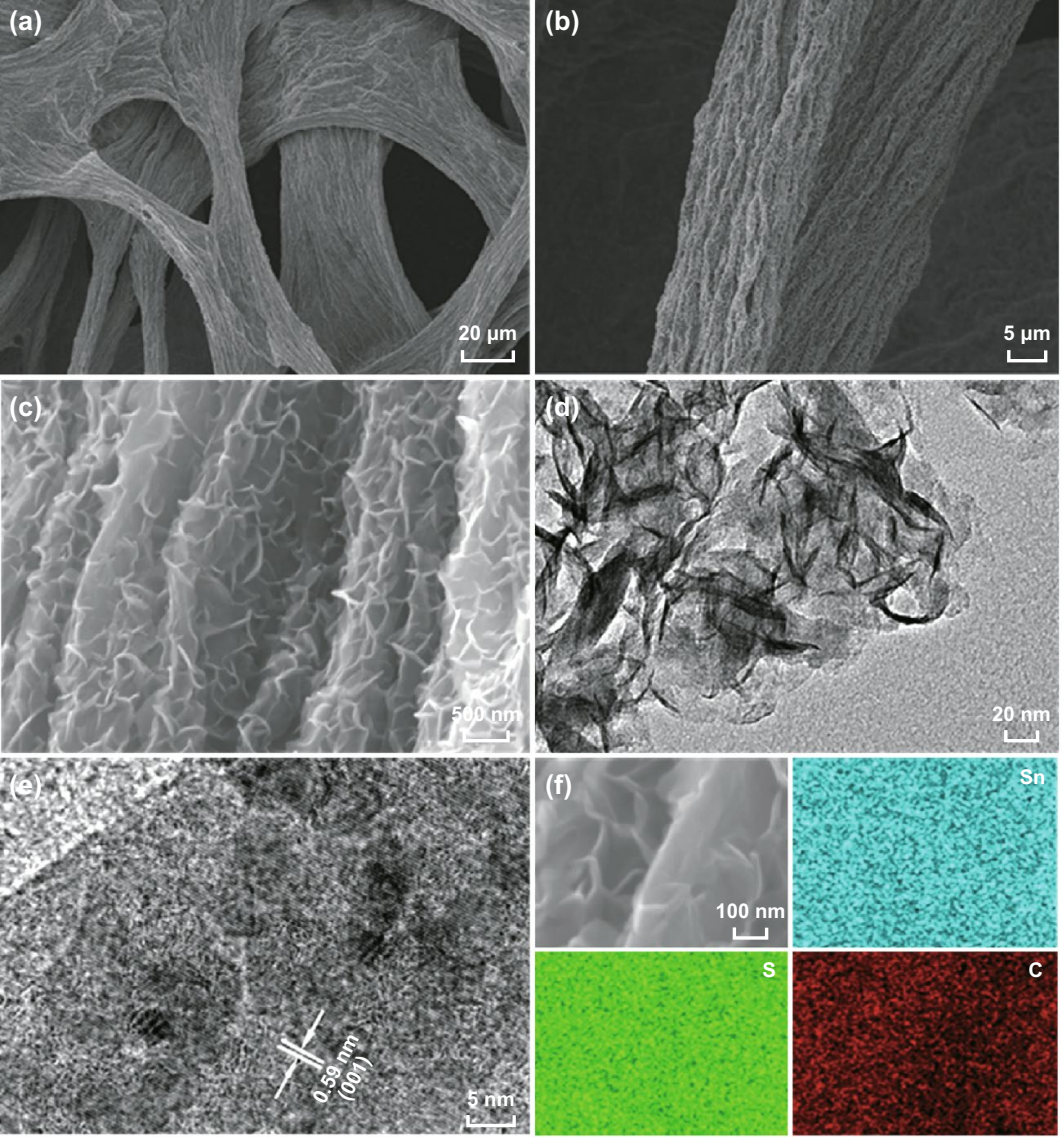
**a**–**c** Low- and high-resolution SEM images, **d** TEM image, **e** HRTEM image, and **f** EDX mapping results of the SnS_2_@rGF fabrics

The strength of graphene fiber and SnS_2_@rGF is shown in Fig. S2. The microstructures of the SnS_2_@rGF fabrics are examined by SEM, TEM, HRTEM, and EDX mapping. The low-magnified SEM image clearly depicted a porous and continuous network formed by the stacked and fused rGO fibers (Fig. 2a, b). The lateral diameter of rGO fibers was about several micrometers. Closer observation shows that there are lots of wrinkles on the surface of each fiber, which is beneficial for the decoration of guest materials. The high-magnified SEM image displayed that densely packed SnS_2_ nanosheets with thickness of several nanometers have successfully covered onto the entire surface of rGF, as seen in Fig. 2c. The corresponding EDS mapping demonstrates the uniform distribution of Sn, S, and C elements within the hybrid fabrics, further verifying the successful composition of SnS_2_ with rGF (Fig. 2f). Owing to in situ transformation from SnO_2_ nanocrystals, it is reasonable to speculate that the strong interfacial interactions are preserved between SnS_2_ nanosheets and rGF, which is beneficial for the interfacial ion transport. The detailed microstructure was further characterized by TEM, which shows that the thickness of the SnS_2_ nanosheets is in the range of 2–5 nm, indicating a short ions diffusion length. HRTEM revealed a single crystalline character of the SnS_2_ nanosheets. The interplanar spacing is 0.59 nm, matching well with the (001) plane of 2T-type layered structure SnS_2_.

**Fig. 3:**
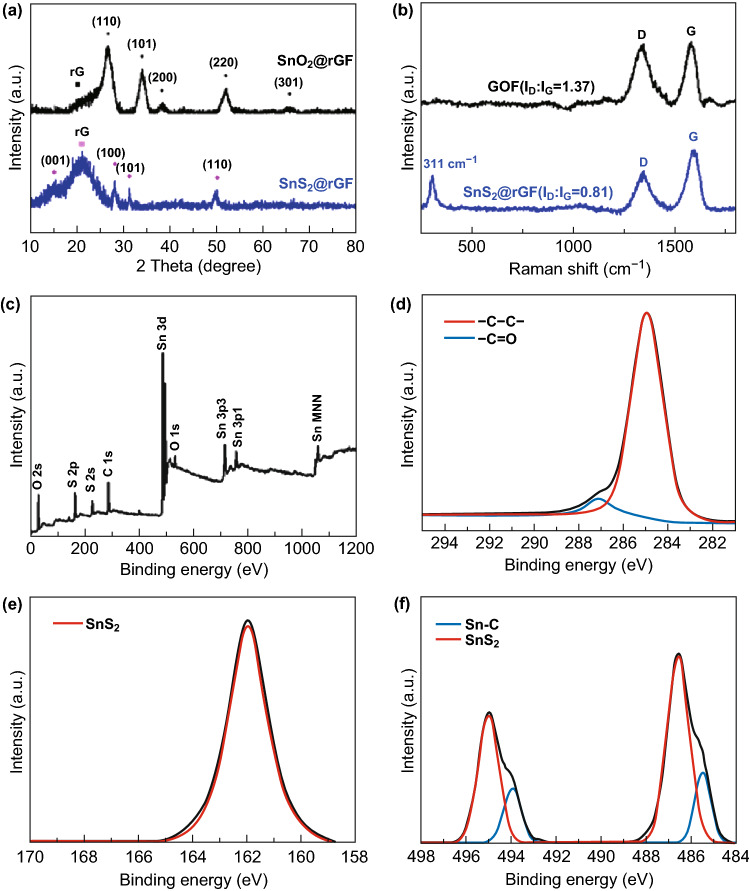
**a** Wide-angle XRD patterns of the SnO_2_@rGF and SnS_2_@rGF fabrics, **b** Raman spectra of the GOF and SnS_2_@rGF. **c–f** XPS spectra of the SnS_2_@rGF fabrics, **c** survey, **d** C 1 *s* region, **e** S 2*p* region, and **f** Sn 3*d* narrow scan spectra

XRD measurements were employed to confirm the phase structure of SnO_2_@rGF and SnS_2_@rGF fabrics. The diffraction patterns of the SnS_2_@rGF fabrics can be readily indexed as standard 2T-type layered structure (JCPDS card No. 23-0677) with no significant impurities peaks, as displayed in Fig. 3a (blue curve). The broad diffraction peak centered at 20.89° could be assigned to rGF. There is no obvious diffraction peaks at 26° and 34° which represent (110) and (101) of SnO_2_ crystal in the SnS_2_@rGF pattern [45]. This means SnO_2_ has completely converted into SnS_2_ via sulfurization. TGA profile in Fig. S6 shows that the mass content of SnS_2_ in the SnS_2_@rGF fabrics is about 67.2 wt%.

To prove that the graphene fiber can improve the electrical conductivity of electrode, the electrical conductivities of GOF, rGF, and SnS_2_@rGF were tested, which are 0.000345, 347.2 and 510.2 S m^−1^, respectively. Due to the loose stacking of graphene layers in the fiber, the conductivity of rGF is low than that of SnS_2_@rGF. Raman spectra of the SnS_2_@rGF fabrics are presented in Fig. 3b. There are two characteristic bands observed at ~ 1330 and 1580 cm^−1^ [46], assigning to D band and G band of graphene, respectively [47]. The *I*_D_/*I*_G_ ratio of SnS_2_@rGF is 0.81, much less than that of GOF (1.37). The relative low *I*_D_/*I*_G_ ratio is a clear signal of the decrease of structural defects of rGF, which finally improve the electronic conductivity and mechanical strength of rGF [44]. XPS was used to further explore the surface chemical composition and chemical states of the SnS_2_@rGF. The elements of Sn, S, O, and C are detected in XPS survey spectrum, without other impurities (Fig. 3c). The C 1*s* spectrum in Fig. 3d could be deconvoluted into two peaks centered at 284.80 and 286.88 eV, corresponding to C–C and C=O bonds, respectively. The oxide agents are much less in SnS_2_@rGF than that in GOF (in Fig. S7). The low peak intensity of carbon functional groups suggests a deep reduction of rGF, agreeing well with the Raman results. For the S 2*p* spectrum, one peak is detected at 161.85 eV, which is a characteristic of S 2*p*_3/2_ signal in SnS_2_ (Fig. 3e). Figure 3f displays Sn 3*d* spectrum, where the two peaks centering at 486.47 and 495.00 eV could be ascribed to Sn 3*d*_5/2_ and Sn 3*d*_3/2_ (with a spin energy separation of 8.40 eV), respectively, which is a characteristic of Sn^4+^ in SnS_2_ [48]. Besides, there are two other peaks at 485.38 and 493.84 eV, which are close to the Sn 3*d*_5/2_ and Sn 3*d*_3/2_ peaks of SnS [49, 50]. However, there is no obvious diffraction peaks of SnS phases were detected by XRD [45]. Another possible reason for this phenomenon is that the Sn atoms interact with C atoms in the graphene fibers, which is conformed to the C 1*s* spectra.

**Fig. 4:**
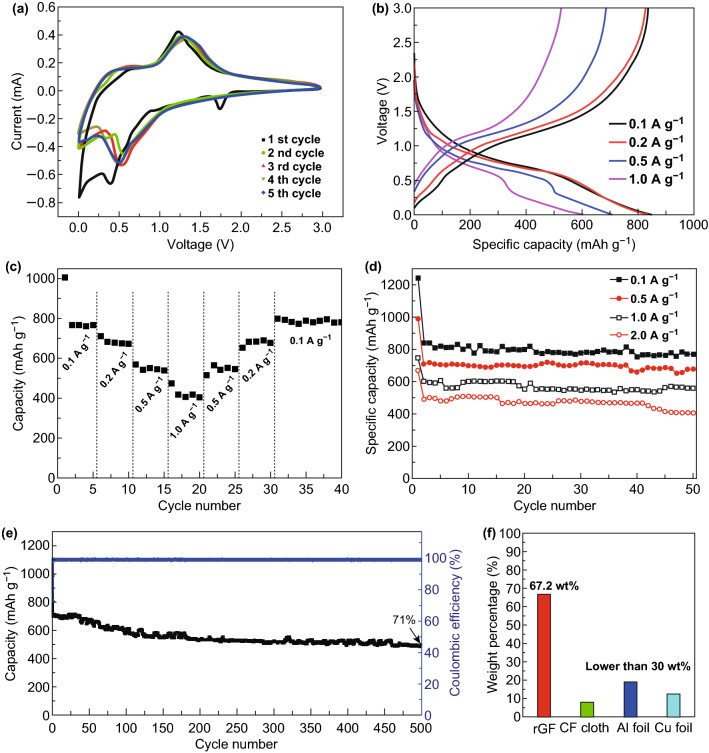
**a** Cyclic voltammograms, **b** the 2nd discharge/charge curves at different current rates and **c** the rate performances of the SnS_2_@rGF electrode. **d** Rate capability of the SnS_2_@rGF anodes with cycles. **e** Long-term cycle performance of the SnS_2_@rGF electrode at a current rate of 0.5 A g^−1^. **f** Comparison of the estimated weight percentage of SnS_2_ in electrodes using different current collectors, including the commonly employed CF cloth (CeTech, WOS1002), Al foil (18 µm in thickness), Cu foil (9 µm in thickness), and our rGF fabrics

To evaluate the electrochemical properties of the flexible SnS_2_@rGF fabrics, we first performed CV tests at 0.25 mV s^−1^, as displayed in Fig. 4a. There are three sharp peaks observed at 1.76, 0.44 and 0.02 V in the first cathodic process, and a broad peak ranging from 1.2 to 0.9 V [51]. The peak at 1.76 V is commonly assigned to the processing that Na^+^ inserts into SnS_2_. The broad peak, between 1.2 and 0.9 V, is commonly assigned to the conversion reaction accompanied by the formation of metallic Sn and Na_2_S [52], while the cathodic one between 0.44 and 0.02 V could be attributed to the formation of the solid electrolyte interphase (SEI) in the first cycle [53, 54] and the metallic reaction. In subsequent cycles, the peak at 0.44 V is replaced by a peak at 0.55 V, suggesting the well reversible and predominant storage reactions. There is a peak at around 0.02 V indicating the formation of Na_*x*_Sn alloy [23, 55]. In this work, the irreversible capacity mainly occurred in the first cycle. The Na_*x*_Sn alloy forming in discharge process would well decompose in charge process, which is proved at a later section. The moderate peak relating to decomposition is at 0.6–0.8 V in charge process. During the anodic process, a small oxidation peak at 0.4 V is attributed to the conversion reaction from Na_*x*_Sn to Sn. The peak at 1.25–1.3 V is dominated by the reaction from Sn to SnS_2_ [56]. Well overlapping of CV curves after the first cycle demonstrates the good reversibility of the binder-free SnS_2_@rGF anode.

The galvanostatic discharge–charge profiles of the designed flexible SnS_2_@rGF anode at different current densities are illustrated in Fig. 4b. Apparently, there are two discharge voltage platforms at 1.0–0.6 V and below 0.2 V, with a charge voltage platform between 1.2 and 1.3 V in the curves. The platforms at 1.0–0.6 V and 1.2–1.3 V indicate the cathodic reaction and anodic reaction separately. The platform below 0.2 V is mainly attributed to the formation of Na_*x*_Sn alloy [27, 55, 57], which is more obvious at high current rates. The specific capacities of SnS_2_@rGF anodes were calculated based on the mass of SnS_2_, owing to extremely low sodium ions storage capacities of pure rGF fabrics (less than 15 mAh g^−1^), as seen in Fig. S8a. Even though the current density is low as 0.1 A g^−1^, the rGF delivers the specific capacity of less than 25 mAh g^−1^. As calculated, the cell could deliver specific capacities of 836, 818, 706, 593, and 492 mAh g^−1^ at current rates of 0.1, 0.2, 0.5, 1.0, and 2.0 A g^−1^ at the second cycles, respectively (Fig. 4b). The specific capacity at a low current density (0.1 A g^−1^) is literally close to theoretical value. The Coulombic efficiency is as high as 97.48%, showing the good reversibility and stability of the binder-free anode after formation of SEI film. This kind of cycle and rate performances benefit from the good electron conductivity and porosity of rGF fabrics, which is more accessible for the insertion and diffusion of Na^+^.

To highlight the performances of the flexible SnS_2_@rGF anode, the energy storage properties of the traditional SnS_2_ electrode were also tested. As shown in Fig. S8a, the specific capacities of the traditional SnS_2_ electrode decrease seriously from 1160.96 mAh g^−1^ of the first cycle to 390.62 mAh g^−1^ in the second cycle at a current density of 0.5 A g^−1^, which even less than 200 mAh g^−1^ after three cycles. Furthermore, the flexible SnS_2_@rGF anode exhibits an good rate performance and cyclic stabilities, as shown in Fig. 4c. Remarkably, when the current rate returns to 0.1 A g^−1^ at the 31st cycle, a specific capacity of 798 mAh g^−1^ is recovered, even higher than that of the frontmost cycle.

The cycling performance of SnS_2_@rGF was evaluated at different current rates (Fig. 4d). After 50 cycles, the Na metal||SnS_2_@rGF provides a high capacity of 767, 677, 557, and 406 mAh g^−1^ at current densities of 0.1, 0.5, 1.0, and 2.0 A g^−1^, respectively, exhibiting a stable rates performance. To further explore the promising potential of the flexible SnS_2_@rGF fabrics as flexible anode for SIBs, we carried out the long-term cycling test at a current density of 0.5 A g^−1^, as shown in Fig. 4e. In the first cycle, the Coulombic efficiency is 71.3%, which is caused by the formation of SEI film. To better quantify the capacity loss and exclude the effect of SEI formation, the specific capacity at the second cycle was used as the baseline (706 mAh g^−1^) [58]. The percentage of surplus capacity at 50th, 100th, 200th, and 500th cycle is 95.89% (677 mAh g^−1^), 82.57% (583 mAh g^−1^), 76.20% (538 mAh g^−1^), and 70.82% (500 mAh g^−1^), respectively. The Coulombic efficiency maintains an ultrahigh level of 95% in the second cycle and as high as ~ 100% in the follows. In our opinion, this is due to the good strength of graphene fiber (~ 65 MPa), large interlayer of SnS_2_ (0.59 nm), in situ sulfurization of SnO_2_–SnS_2_, and the formation of a stable SEI after the first cycle. The good mechanical strength of graphene fiber could ensure a stable electrode conductive scaffold, to prohibit the agglomerations of active materials during electrochemical cycles. The larger interlayer of SnS_2_ could endow a fast electrochemical kinetics during Na^+^ insertion and extraction. Thirdly, in situ sulfurization of SnO_2_–SnS_2_ could introduce a strong interface interaction between SnS_2_ nanosheets and graphene layer, further inhibiting the active materials aggregation. And also, the ultrathin and quasi-vertical SnS_2_ nanosheets could expose all of active sites in electrolyte, thus leading to a fully formation of SEI in the first cycle and finally to give a higher Coulombic efficiency onwards.

In order to further highlight the ultralight character of flexible rGF fabrics, we also make a comparison of weight percentage of SnS_2_ in different electrodes by hypothesizing that the same weight of SnS_2_ was deposited onto the common current collectors such as CF cloth, Al foil, and Cu foil in the same electrode diameter (12 mm). As shown in Figs. 4f, S2 and Table S1, clearly, the mass loading of the active materials in other electrode configuration could not even reach up to 30 wt%, which is much less than that of the developed rGF fabrics (67.2 wt%) in this work (the highest value is 52.5 wt% in the literature). Although some of reported specific capacities are higher than that of our reported values, however, if including the mass of current collector, polymer binders and conductive additives, the effective specific capacities are less than 200 mAh g^−1^, highlighting the ultralight nature of the rGF fabrics and promising potentials in energy storage devices.

The electrochemical impedance spectra (EIS) of the SnS_2_@rGF half cell were measured to fully understand the role of rGF. The typical Nyquist plots characteristic of a semicircle in the high-frequency region and a slope line in the low-frequency region are corresponding to the charge transfer resistance *R*_ct_ and the Warburg impedance *Z*_w_ dependent on the Na^+^ diffusion in the electrodes, separately [59, 60]. Noticeably, the semicircle after galvanostatic charge–discharge is much smaller than that of fresh cell (Fig. S8c), suggesting a hysteretic activation process of anode materials. The semicircles at 50th cycle and 100th cycle showed similar shapes and nearly overlapped with each other, reasonable indicating the structural stability of the conductive rGF.

To fully understand the electrochemical reaction during cycles, SEM and XPS techniques were further employed to examine the microstructures and chemical states of the flexible SnS_2_@rGF electrode after 100 cycles, as illustrated in Figs. S9 and S10. Clearly, a layer of SEI instead of SnS_2_ nanosheets was observed on the surface of the flexible electrode. The structure of rGF was preserved with no serious aggregation. EDX results demonstrate that the uniform distributions of Sn and S elements were kept well. However, a strong Na signal was observed as shown in Fig. S9d, indicating that Na ions are not full extracted from SnS_2_ structure and the formation of SEI. The XPS results after 100 cycles are shown in Fig. S10, only characteristic XPS peaks of SnS_2_ and SnS, and an obvious peak centered at 1071.12 eV corresponding to Na^+^ were observed. No signals of Na^0^ were detected, indicating that the Na_*x*_Sn alloys were well decomposed during recharging and SnS_2_ phases were recovered. This is the reason for continuous high Coulombic efficiency and high utilization of active materials, which contributes to the stable rate ability and good cycle performance.

## Conclusions

In summary, we firstly fabricated SnS_2_@rGF hybrid fabrics via hydrothermal and sulfurization reactions as binder-free electrode for SIBs. The designed electrode possesses several merits, such as highly porous and forming a continuous conductive network via the interfused graphene fibers, which could greatly improve the ions diffusion, electron transportation, and accommodation of volume expansion. As well, the in situ formed interfacial interaction of SnS_2_ nanosheets and graphene would yield good structural stability and interfacial charge transfer. Most importantly, employing rGF fabrics as flexible current collector could significantly increase the effective mass loading of the active materials in electrode because of its ultralight and porous nature, finally leading to good electrochemical performances and higher real energy density according to the weight of whole electrode. The binder-free SnS_2_@rGF hybrid fabrics deliver specific capacity of 767, 677, 557 and 406 mAh g^−1^ at current rates of 0.1, 0.5, 1.0 and 2.0 A g^−1^, respectively, at 50th cycle. Even at a current density of 0.5 A g^−1^, it can still provide a capacity of 500 mAh g^−1^ after 500 cycles with Coulombic efficiency of ~ 100%. In general, the results highlight that the rGF is a promising framework for using as binder-free electrode of SIBs.

## Electronic supplementary material

Below is the link to the electronic supplementary material.

Supplementary material 1 (PDF 723 kb)

Supplementary material 2 (MP4 16343 kb)
