# Supplementary material for: Rational Design of Layered SnS2 on Ultralight Graphene Fiber Fabrics as Binder-Free Anodes for Enhanced Practical Capacity of Sodium-Ion Batteries
Source: Nanomicro Lett. 2019 Aug 3;11:66. doi: 10.1007/s40820-019-0297-6 (PMC7770950; doi:10.1007/s40820-019-0297-6)
Supplement: Supplementary file 1 — Supplementary material 1 (PDF 723 kb) [file 40820_2019_297_MOESM1_ESM.pdf]

Supporting Information for

Rational Design of Layered SnS_2 on Ultralight Graphene Fiber Fabrics as Binder-Free Anodes for Enhanced Practical Capacity of Sodium-Ion Batteries

Zongling Ren[1], Jie Wen[1], Wei Liu[1], Xiaoping Jiang[1], Yanheng Dong[3], Xiaolong Guo[1], Qiannan Zhao[1], Guipeng Ji[1], Ronghua Wang[3], Ning Hu[1], Baihua Qu[2,] *, Chaohe Xu[1, 4,] *

[1]College of Aerospace Engineering and The State Key Laboratory of Mechanical Transmissions, Chongqing University, Chongqing 400044, People's Republic of China

[2]Pen-Tung Sah Institute of Micro-Nano Science and Technology, Xiamen University, Xiamen 361005, People's Republic of China

[3]College of Materials Science and Engineering, Chongqing University, Chongqing 400044, People's Republic of China

[4]MOE Key Laboratory of Low-grade Energy Utilization Technologies and Systems, CQU-NUS Renewable Energy Materials & Devices Joint Laboratory, Chongqing University, Chongqing 400044, People's Republic of China

*Corresponding authors. E-mail: xche@cqu.edu.cn (Chaohe Xu); bhqu@xmu.edu.cn (Baihua Qu)

Supplementary Figures and Table

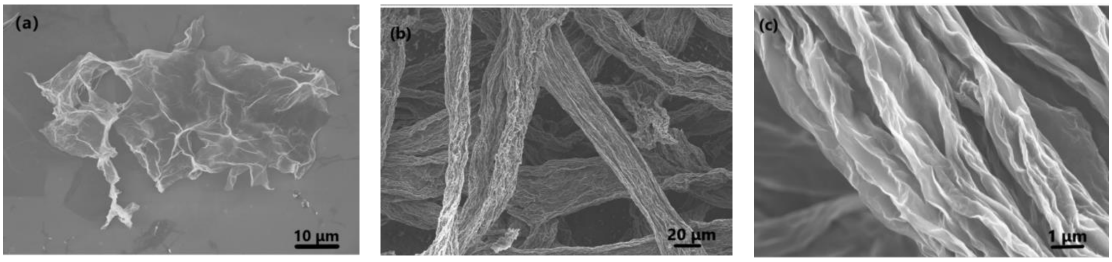

Fig. S1 SEM images of **a** GO, and **b, c** rGF

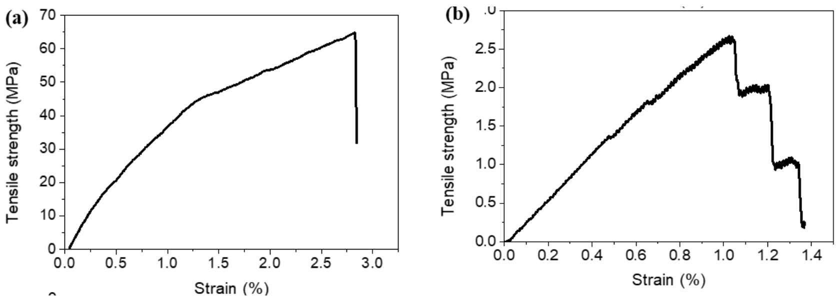

Fig. S2 The tensile strength of **a** rGF fiber and **b** rGF fabric. In the tensile experiment, the length of rGF fiber is 0.5 mm, and the size of fabric is 20 mm long, 5 mm wide, 14 μm thick

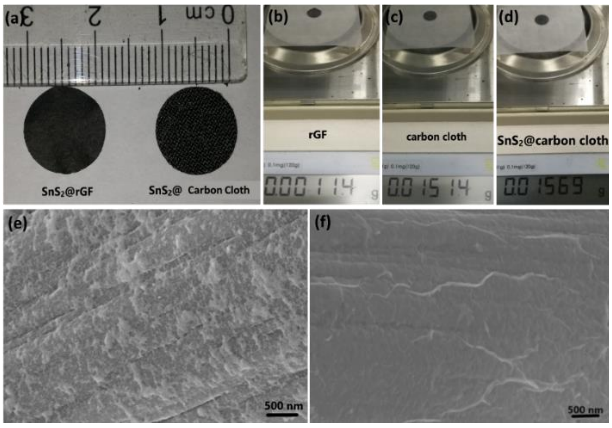

Fig. S3 a Optical photo of SnS_2@rGF and SnS_2@carbon cloth electrode. The weight of **b** rGF and **c** carbon cloth electrode, **d** SnS_2@carbon cloth electrode. SEM photos of the **e** SnO_2@carbon cloth and **f** SnS_2@carbon cloth. In **Fig. S3a–d**, the diameter of all samples is 12 mm. It can be told obviously whether there is SnS_2 attached; rGF fabric samples are much lighter than the carbon cloth samples

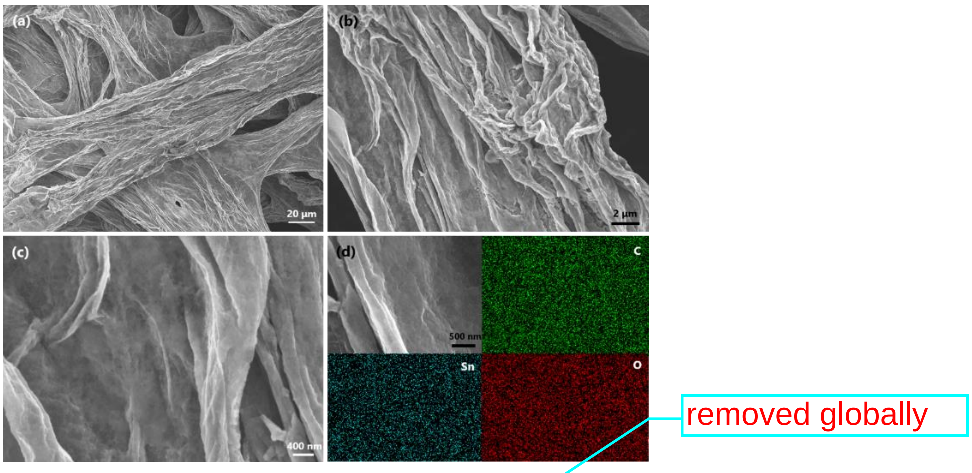

Fig. S4 a-c SEM images, **d** elemental mapping images of the SnO_2@rGF

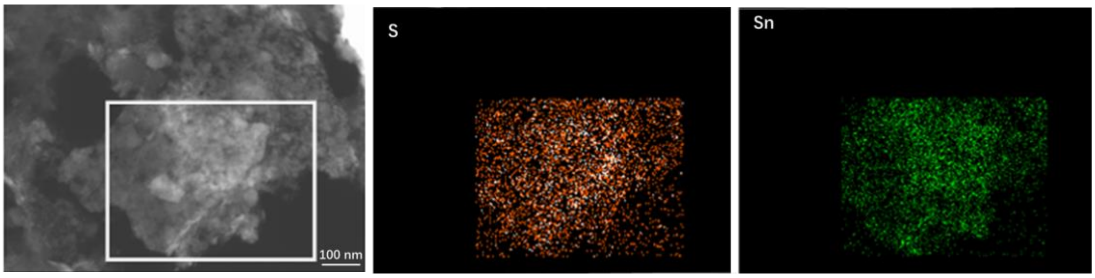

Fig. S5 EDX-STEM images of SnS_2@rGF

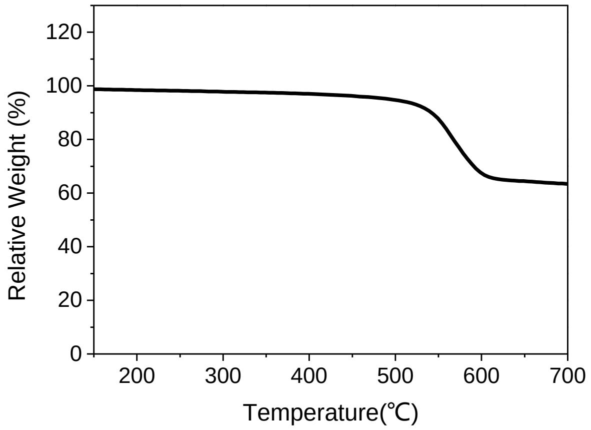

Fig. S6 Thermogravimetric analysis (TGA) prifile of SnS_2@rGF composites

In O_2 atmosphere, the SnS_2@rGF was heated to 700 °C, then turn into SnO_2. The remaining mass was 60.05 wt%, the mass percentage of SnS_2 was calculated by Eq. S1:

$$\text{Mass Percentage} = \frac{\frac{M_{SnS_2}}{M_{SnO_2}} * 0.6005}{1} * 100\% = 67.2\% \qquad \text{(S1)}$$

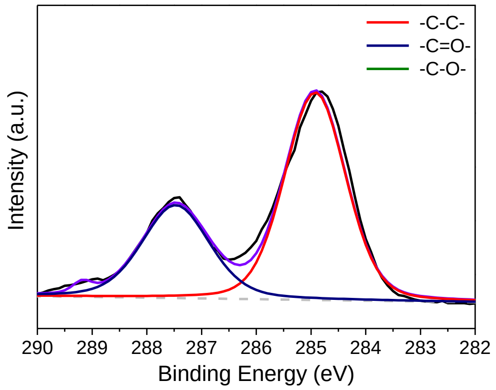

Fig. S7 XPS spectra C1s region of GOF

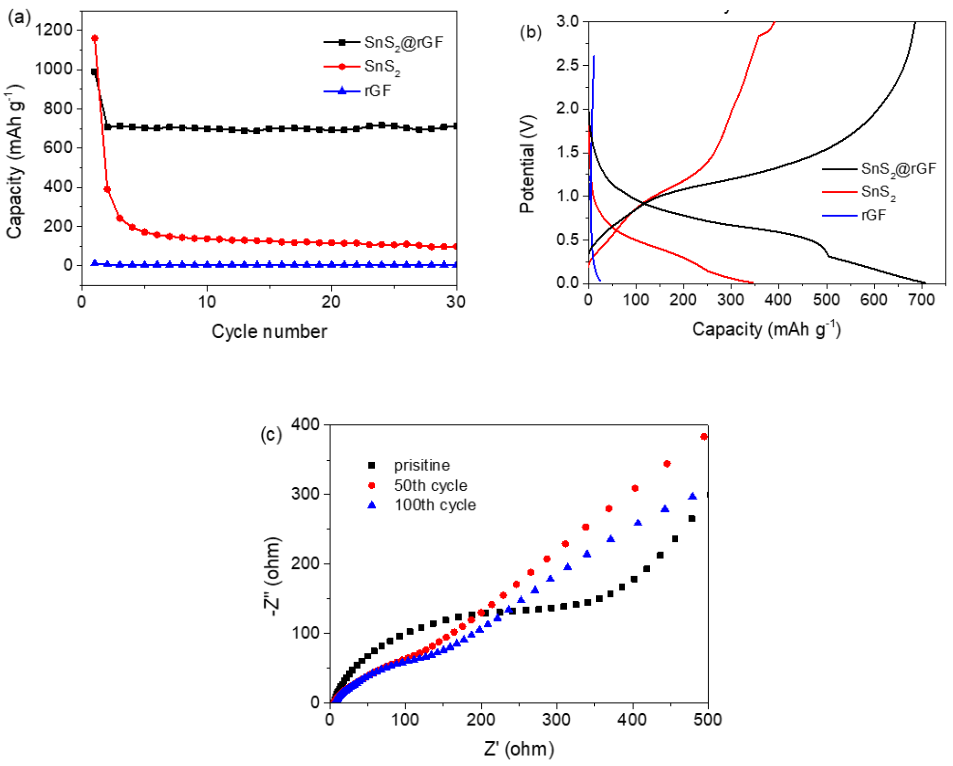

Fig. S8 a cycle rate and **b** 2rd discharge-charge curves of the SnS_2@rGF, SnS_2, and rGF electrode at a current rate of 0.5 A g^{-1}. **c** pristine, 50th cycle, and 100th cycle in a frequency range of 100 kHz to 0.1 Hz with AC voltage amplitude of 5 mV

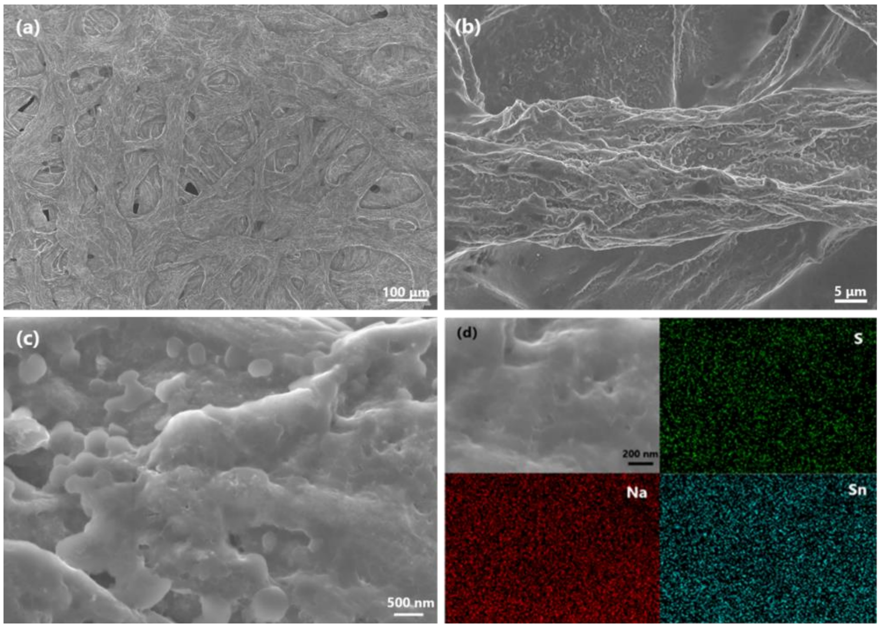

Fig. S9 a-c SEM images, and **d** elemental mapping images of the SnS_2@rGF after 100 cycles

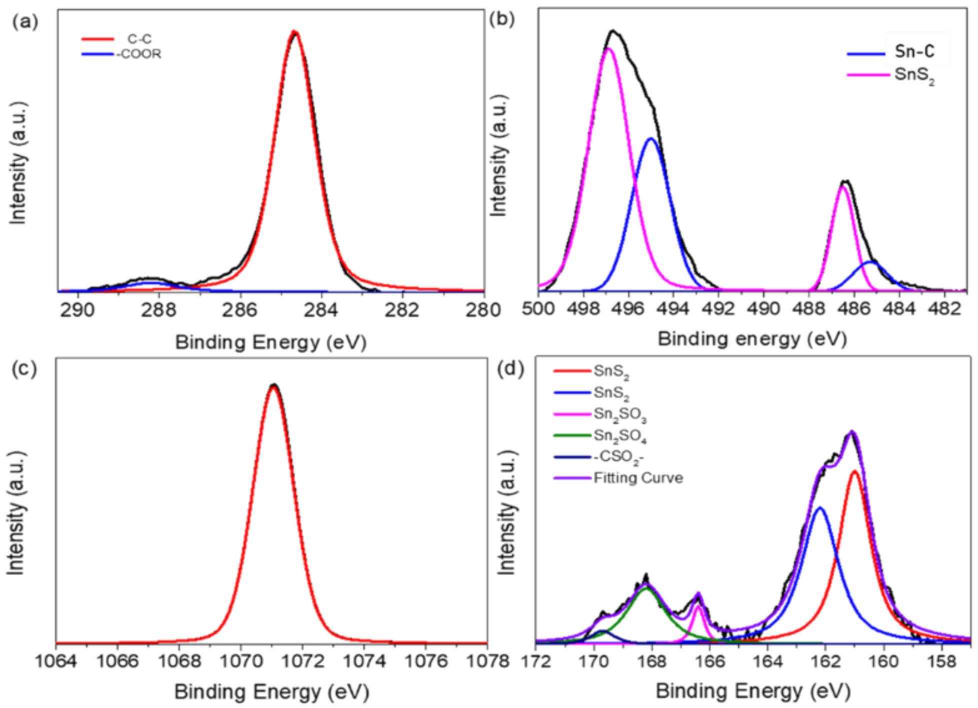

Fig. S10 XPS spectra of SnS_2@rGF composites: **a** C 1s region, **b** S 2p region, **c** Na 1s region and **d** Sn 3d narrow scan spectra

Figure S10b shows that there is no peak corresponding to chlorine, so Na^+ doesn't exist with a form of Sodium Perchlorate.

Table S1 Comparison of sodium storage performance of different anode materials at current rate of 0.2 A g^{-1}

Refs.	Active Material	Substrate	If separated Current Collector	Current Collector	Electrolyte	Active Material Weight Percentage (%, including current collector)	Reversible Specific Capacity before 50 cycles (mAh g^{-1})	Specific Capacity (mAh g^{-1}, including current collector)
[S1]	SnS_2	Graphene, CNTs	yes	Copper foil	1 M $NaClO_4$ in PC and EC (1:1 by volume) along with 5 wt% of FEC	32.24 wt%	600	194
[S2]	SnS_2	Carbon hollow structure	yes	Copper foil	1 M $NaClO_4$ in PC and EC (1:1 by volume) along with 5 wt% of FEC	24.82 wt%	696	173
[S3]	SnS_2	Sea-sponge structure	yes	Copper foil	1 M $NaClO_4$ in PC and EC (1:1 by volume) along with 5 wt% of FEC	21.17 wt%	530	112
[S4]	SnS	CNTs	yes	Copper foil	1 M $NaClO_4$ in PC and EC (1:1 by volume) along with 5 wt% of FEC	24.82 wt%	435	108
[S5]	SnS_2	graphene	yes	Ni foam	1 M $NaClO_4$ in PC and EC (1:1 by volume) along with	No collector density data	582	/

					5 wt% of FEC			
[S6]	SnS_2	MWNTs, C	yes	Ni foam	1 M $NaClO_4$ in PC and EC (1:1 by volume) along with 5 wt% of FEC	No collector density data	910 (0.1 A g^{-1})	/
[S7]	SnS_2	Carbon paper	no	/	1 M $NaClO_4$ in PC and EC (1:1 by volume) along with 5 wt% of FEC	Not mentioned	1006	/
[S8]	SnS_2	Graphene nanoribbons	no	/	1 M $NaClO_4$ in PC and EC (1:1 by volume) along with 5 wt% of FEC	52.5 wt%	1032	542
[S9]	SnS	Carbon nanofiber	Free-Standing<br>1 M $NaClO_4$ in PC and EC (1:1 by volume) along with 5 wt% of FEC				444	/
[S10]	SnS_2	graphene foam	No	/	1 M $NaClO_4$ in PC and EC (1:1 by volume) along with 5 wt% of FEC	Not mentioned	900	/
This work	SnS_2	rGF fabric	no	/	1 M $NaClO_4$ in PC and EC (1:1 by volume) along with 5 wt% of FEC	67.2 wt%	802	539

All these data in this blank is found in original papers. The radium of electrode which is not mentioned in some papers is postulated as 12 mm, which actually has no influence on the calculation. The specific capacity (including current collector) is calculated on the basis of Eq. S2:

$$\text{Specific Capacity} = \frac{\text{Reversible Specific Capacity Mentioned in Article}}{m_{active\ material} + m_{binder} + m_{collector}} \quad \text{(S2)}$$

Comparison of the estimated mass loading based on commonly employed CF cloth (CeTech, WOS1002), Al foil (15 μm), Cu foil (9 μm), and our rGF fabrics.

Supplementary References

[S1] J. Cui, S. Yao, Z. Lu, J.Q. Huang, W.G. Chong, F. Ciucci, J.K. Kim, Revealing pseudocapacitive mechanisms of metal dichalcogenide SnS_2/graphene-CNT aerogels for high-energy Na hybrid capacitors. Adv. Energy Mater. **8**, 1702488 (2018) . https://doi.org/10.1002/aenm.201702488

[S2] Y. Liu, X.Y. Yu, Y. Fang, X. Zhu, J. Bao, X. Zhou, X.W. Lou, (David). Confining SnS_2 ultrathin nanosheets in hollow carbon nanostructures for efficient capacitive sodium storage. Joule **2**(4), 725–735 (2018) . https://doi.org/10.1016/j.joule.2018.01.004

[S3] S. Chen, K. Xing, J. Wen, M. Wen, Q. Wu, Y. Cui, Hierarchical assembly and superior sodium storage properties of a sea-sponge structured C/SnS@C nanocomposite. J. Mater. Chem. A **6**(17), 7631–7638 (2018) . https://doi.org/10.1039/c8ta00833g

[S4] P. He, Y. Fang, X.Y. Yu, X.W.D. Lou, Hierarchical nanotubes constructed by carbon-coated ultrathin sns nanosheets for fast capacitive sodium storage. Angew. Chem. Int. Ed. **56**(40), 12202–12205 (2017) . https://doi.org/10.1002/anie.201706652

[S5] Y. Zhang, P. Zhu, L. Huang, J. Xie, S. Zhang, G. Cao, X. Zhao, Few-layered SnS_2 on few-layered reduced graphene oxide as Na-ion battery anode with ultralong cycle life and superior rate capability. Adv. Funct. Mater. **25**(3), 481–489 (2015) . https://doi.org/10.1002/adfm.201402833

[S6] Y. Zhao, B. Guo, Q. Yao, J. Li, J. Zhang, K. Hou, L. Guan, A rational microstructure design of SnS_2-carbon composites for superior sodium storage performance. Nanoscale **10**(17), 7999–8008 (2018) . https://doi.org/10.1039/c8nr01783b

[S7] J.G. Wang, H. Sun, H. Liu, D. Jin, R. Zhou, B. Wei, Edge-oriented SnS_2 nanosheet arrays on carbon paper as advanced binder-free anodes for Li-ion and Na-ion batteries. J. Mater. Chem. A **5**(44), 23115–23122 (2017) . https://doi.org/10.1039/c7ta07553g

[S8] Y. Liu, Y. Yang, X. Wang, Y. Dong, Y. Tang, Z. Yu, Z. Zhao, J. Qiu, Flexible paper-like free-standing electrodes by anchoring ultrafine SnS_2 nanocrystals on graphene nanoribbons for high-performance sodium ion batteries. ACS Appl. Mater. Interfaces **9**(18), 15484–15491 (2017) . https://doi.org/10.1021/acsami.7b02394

[S9] J. Xia, L. Liu, S. Jamil, J. Xie, H. Yan et al., Free-standing SnS/C nanofiber anodes for ultralong cycle-life lithium-ion batteries and sodium-ion batteries. Energy Storage Mater. **17**, 1–11 (2018) . https://doi.org/10.1016/j.ensm.2018.08.005

[S10] D. Chao, P. Liang, Z. Chen, L. Bai, H. Shen et al., Pseudocapacitive Na-ion storage boosts high rate and areal capacity of self-branched 2D layered metal chalcogenide nanoarrays. ACS Nano **10**(11), 10211–10219 (2016) . https://doi.org/10.1021/acsnano.6b05566
